# A “Third Wheel” Effect in Health Decision Making Involving Artificial Entities: A Psychological Perspective

**DOI:** 10.3389/fpubh.2020.00117

**Published:** 2020-04-28

**Authors:** Stefano Triberti, Ilaria Durosini, Gabriella Pravettoni

**Affiliations:** ^1^Department of Oncology and Hemato-Oncology, University of Milan, Milan, Italy; ^2^Applied Research Division for Cognitive and Psychological Science, IEO, European Institute of Oncology IRCCS, Milan, Italy

**Keywords:** decision making, artificial intelligence, ehealth, patient-doctor relationship, technology acceptance, healthcare process, patient-centered medicine

## Abstract

In the near future, Artificial Intelligence (AI) is expected to participate more and more in decision making processes, in contexts ranging from healthcare to politics. For example, in the healthcare context, doctors will increasingly use AI and machine learning devices to improve precision in diagnosis and to identify therapy regimens. One hot topic regards the necessity for health professionals to adapt shared decision making with patients to include the contribution of AI into clinical practice, such as acting as mediators between the patient with his or her healthcare needs and the recommendations coming from artificial entities. In this scenario, a “third wheel” effect may intervene, potentially affecting the effectiveness of shared decision making in three different ways: first, clinical decisions could be delayed or paralyzed when AI recommendations are difficult to understand or to explain to patients; second, patients' symptomatology and medical diagnosis could be misinterpreted when adapting them to AI classifications; third, there may be confusion about the roles and responsibilities of the protagonists in the healthcare process (e.g., Who *really* has authority?). This contribution delineates such effects and tries to identify the impact of AI technology on the healthcare process, with a focus on future medical practice.

## Introduction

In the last few years, Artificial Intelligence (AI) has been on the rise, and some think that this technology will define the contemporary era as automation and factory tools defined the industrial revolutions, or as computers and the web characterized recent decades (1–3). These technologies, based on machine learning, promise to become more than simple “tools”; rather, they will be interlocutors of human operators that can help in complex tasks involving reasoning and decision making. The expression “machine learning” refers to a branch of computer science devoted to developing algorithms able to learn from experience and the external environment, improving performance over time (4–6). More specifically, algorithms are able to detect associations, similarities, and patterns in data, allowing predictions to be made on the likelihood of uncertain outcomes.

AIs and machine learning are present in a number of commonly used technologies, such as email, social media, mobile software, and digital advertising. However, the near future of AI is not that it will continue to work outside of the end users' awareness, as it mostly does nowadays; on the contrary, AI promises to become an active collaborator with human operators in a number of tasks and activities. AIs are able to analyze enormous quantities of data of various contents and formats, even where it is dynamically changing (Big Data). AIs identify associations and differences between data and provide human operators with outputs that are impossible to achieve by humans alone, at least in the same amount of time.

An example of such outputs are medical diagnoses and the identification of therapy regimens to be administered to patients. Health professionals (physicians especially) will increasingly interact with AIs to get information on their patients that will hopefully be more exact, specific, and based on objective data (7–9). Diagnostic decision support could be considered the main application area for AI-based innovation in medical practice (10, 11). Basically, machine learning devices are trained to classify stimuli based on initial examples. For instance, tumor types can be identified by the comparison of patient's TAC with information coming from scientific literature (12, 13); the same can be done with pictures of skin lesions (14), optical coherence tomography in the case of sight diseases (15–17), or the integration of clinical observations and medical tests for other diseases (18, 19). While diagnosis is recognized by many as the main area for AI implementation in medicine and healthcare, others could be envisaged, as summarized in Table 1.

**Table 1:** A resume of the main areas for AI implementation in healthcare and medicine.

**AI function in healthcare**	**Description; AI is…**	**Examples**
Diagnosis	Employed as a diagnostic support tool; it analyzes clinical/pathological data to identify the disease	(11, 16, 20, 21)
Treatment (identification)	Involved in identification of treatment, often patient-specific solutions (genomics, precision medicine); it could participate in providing early interventions to delay the onset of chronic conditions (pre-emptive medicine)	(22–25)
Health management/patient engagement	Featured in devices that collect data on patient health status and provide recommendations for everyday care (eHealth, Digital Therapeutics, Ambient Intelligence)	(26–28)
Health Systems organization support/simulation	Used in agent-based simulations that model care coordination capabilities, providing insights for organizational improvements	(29, 30)

However, the study of AI in healthcare in its social-psychological aspects is still an underrepresented area. One important field is that of “Explainable Artificial Intelligence” (commonly abbreviated in XAI), namely the research on AI's transparency and ability to explain its own elaboration processes. The American Agency for Advanced Research Projects for Defense (DARPA) launched a program on XAI (31), and the European Parliament demands a “right to explanation” in automated decision making (32). Indeed, one issue with AI implementation in professional practice regards the fact that it is supposed to be used by non-professionals: doctors, marketers, or military personnel are not expected to become experts in informatics or AI development, yet they will have to interact with artificial entities to make important decisions in their fields. While one could easily agree with the analyses and outputs of AIs, *trusting them* and taking responsibility for decisions that will affect the “real world” is no easy task. For this reason, XAI is identified by many scholars as a priority for technological innovation. Miller and colleagues (33, 34) maintain that AI developers and engineers should turn to social sciences in order to understand what is an explanation, and how it could be effectively implemented within AIs' capacities. For example, Vellido (35) proposed that AIs learn to make their processes transparent via visual aids that help a human user to understand how a given conclusion has been reached; Pravettoni and Triberti (36) highlighted that explanation is rooted in interaction and conversation, so that a complete, sophisticated XAI would be reached when artificial entities were able to communicate with human users in a realistic manner (e.g., answering questions, learning basic forms of perspective-taking, etc.). In any case, besides working on AI-human interfaces, another field of great interest is that of AI's impact on professional practice or the prediction of possible organizational, practical, and social issues that will emerge in the context of implementation.

Though the contribution of AIs to medical practice is promising, their impact on the clinician-patient relationship is still an understudied topic. From a psychosocial point of view, it is possible that new technologies will influence the relationship between clinicians and patients in several ways. Indeed, the introduction of AI into the healthcare context is changing the ways in which care is offered to patients: the information given by AIs on diagnosis, treatment, and drugs will be used to make decisions in any phase of the healthcare journey (e.g., choices on treatment or lifestyle changes, deciding to inform relatives of one's health status, communication of bad news).

According to a patient-centered perspective, such care choices should be made by the patient and the doctor within a mutual collaboration, which points to the popular concept of *shared decision making*. This concept has become fundamental in the debate on patient-centered approaches to care, with the number of scientific publications on the subject rising more than 600% from 2000 to 2013 (37). Reviews show that the communication process and relationship quality among doctors and patients has a significant effect on patients' well-being and quality of life, so that the proper communication style can alleviate the traumatic aspects of illness (38–40). However, when prefiguring the adoption of AIs participating in diagnosis and therapy identification, it is possible that the same concept of “shared decision” should be updated, taking into consideration the contribution of artificial entities.

## Who to Share Decision Making With?

Shared decision making has been proposed as an alternative paradigm to the “paternalistic” one (41, 42). The latter model dominated disease-centered medicine, with the physician being authoritative and autonomous, giving recommendations to patients without taking into consideration their full understanding, personal needs, and feelings. While the “paternalistic” physician intended to act in the best interest of the patient, such an approach may be ineffective or counterproductive in the end, because the patient may not understand nor follow the recommendations (43). Shared decision making is a process by which patients and health professionals discuss and evaluate the options for a particular medical decision, in order to find the best available treatment that is based on knowledge that is accessible and comprehensible for both and satisfies both needs (44–46). During this process, the patient is made aware of diagnostic and treatment pathways, as well as of related risks and benefits; also, the patient's point of view is taken into consideration in terms of preferences and personal concerns (47–49).

In other words, shared decision making entails a process of communication and negotiation between the health professional and the patient in which both medical information (e.g., diagnosis, therapy, prognosis) and patient's concerns (e.g., doubts and request for clarification, lifestyle changes, worries for the future, etc.) are exchanged.

In the near future, where AI is expected to take a role in medical practice, it is important to understand its influence on shared decision making and on the patient-doctor relationship as a whole. In most of the health systems around the world, the patient has the right to be informed about which tools, resources, and approaches are being employed to treat his or her case; a patient will have to know that the diagnosis or even the medical prescriptions first came out of a machine, not through the human doctor's effort. Presumably, just the knowledge of the presence of a “machine” in the healthcare process could influence the attitudes of doctors and patients: from a psychological point of view, attitudes toward something may develop before any direct experience of it (e.g., as a product of hearsay, social norms, etc.), and influence subsequent conduct (50). Indeed, while medicine itself is inherently open to innovation and technology, some health professionals harbor negative attitudes toward technology for care (51, 52), the main reasons being the risk they feel for patient de-humanization (53, 54) or the fear that tools they are not confident in mastering may be used against them in medical controversies (55). Similarly, patients who do not feel confident in using technology (“computer self-efficacy”) benefit less from eHealth resources than other patients who do feel confident (56, 57), and technological systems for healthcare are not expected to work as desired if development is not tailored to users' actual needs and context of use (26). Though these data regard types of technologies different from AIs, they clearly show that technological innovation in the field of health is hardly a smooth process. While it is clear how the “technical” part of medicine (e.g., improving diagnosis correctness) would benefit from AIs and machine learning devices, their impact on the patient-doctor relationship is mostly unknown.

Furthermore, with the development of eHealth (58, 59) and the diffusion of interactive AIs as commonly used tools (e.g., home assistants), it is possible that chronic patients (e.g., patients with obesity, arthritis, anorexia, heart disease, diabetes) will be assisted by AIs in their everyday health management. Indeed, for example, the American Food and Drugs Administration (FDA) has made “significant strides in developing policies that are appropriately tailored to ensure that safe and effective technology reaches users” [US FDA (60), p.2], promoting the development of Software as a Medical Device (SaMD), devices that play a role in diagnosis or treatment (not only health or wellness management). FDA-approved digital therapeutics include, for example, reSET developed by PEAR Therapeutics (61), which delivers cognitive-behavioral therapy to patients suffering from substance abuse (62). It is possible that similar future resources will include AIs that directly interact with patients based on natural language processing.

In other words, AIs will not be just a “new app” on doctors' devices, but active interlocutors, able to deliver diagnosis, prognosis, and intervention materials to both the doctor and the patient. According to Topol (7), AI's implementation in care could potentially have positive effects, but this depends mainly on doctors' attitudes: for example, if AI were to take on administrative and technical tasks in medicine, doctors would have the occasion to recover the “lost time” for consultation with and empathic listening to their patients, so to improve shared decision making. In this sense, AI would become an active go-between among care providers and patients.

This considered, it becomes fundamental to understand whether we should expect structural changes in the same context of shared decision making and medical consultation. Will patients interact with AIs directly? Will doctors encounter difficulties and obstacles in adapting their work practices to include technologies able to participate in diagnosis and treatment? Are patient-doctor decisions to be shared with artificial entities too?

At the present time, the scientific literature lacks research data to fully respond to these questions. However, by considering the literature on health providers' reactions to technological innovation and the psychology of medicine, it is possible to prefigure some social-psychological phenomena that could occur in the forthcoming healthcare scenarios in order to prepare to manage undesirable side-effects.

## A “Third Wheel” Effect

In common language, the expression “third wheel” refers to someone who is superfluous with respect to a couple. Typically, the focus of the expression is on this third person, who unintentionally finds him or herself in the company of a couple of lovers and feels excluded and out of place. On the other side of the relationship, the couple may be unaware of the stress caused to the third wheel, or they may feel awkward and uneasy because of the unwanted presence. In other words, a third wheel is someone who is perceived as an adjunct, something unnecessary, who may spoil the mood and negatively influence others' experience. Despite just being a popular idiom, this expression is sometimes used in psychological research to describe relationship issues as experienced by research participants (63): new technology, specifically social media, has been called a third wheel as well because of its possible negative influence on relationship quality (64, 65).

We propose to employ the expression “third wheel” to highlight an emergent phenomenon relating to the implementation of artificial entities in real-life contexts: while technologies become more and more autonomous, able to talk, to “think,” and to actively participate in decision making, their role within complex relationships may be unclear to the human interlocutors, and new obstacles to decision making could arise.

We identified three main ways a third wheel effect may appear in medical consultation aided by artificial intelligence: *decision paralysis, or a risk of delay, “Confusion of the Tongues,” and role ambiguity*. In the next sections, these will be described in detail.

### Decision Paralysis, or a Risk of Delay

As previously stated, current AIs are not transparent in their elaboration processes; that is, their interlocutors may have no clear representation of how AIs have reached a given conclusion: this could generate “trust issues,” especially when important decisions should be taken on the basis of these conclusions. According to Topol and his seminal book *Deep Medicine* (7), one positive consequence for AI implementation in medical practice that we could hope for is giving back time to doctors to reserve to empathic consultation and patient-centered medicine. Indeed, if AIs were to take on technical and administrative tasks in medicine, doctors could devote their attention to patients as individuals and improve the “human side” of their profession. However, we should take into account that doctors using AIs will need to contextualize and justify their role within practice and the relationship with the patient. It could be said that doctors will become “mediators” between their artificial allies and the patients: AI's conclusions and recommendations should be reviewed by the doctor, approved and refined, and explained to the patient, answering his or her questions. On the other side, future technologies could include opportunities for direct interaction between AIs and patients: for example, digital therapeutics, or eHealth applications providing assistance to patients and caregivers in the management and treatment of chronic diseases, could potentially include access to the AI providing diagnosis and therapy guidelines. However, we can foresee that a patient would still need approval and guidance from the human doctor for modifications to the treatment schedule, medication intake, specific changes to lifestyle, and everyday agenda.

Such a “mediation” role could be time-consuming and, at least at an organizational level, generate decision paralysis or delays. Imagine that a hospital tumor board has to make a decision on a patient's diagnosis and only half of the board members agree with the AIs' recommendation; or, that a patient receives an important indication from the AI (e.g., stop taking a medication because wearable devices registered unwanted side effects), but he or she struggles to get in contact with his or her doctor to gain reassurance that this is the right thing to do.

These are examples of the implementation of AIs giving rise to a risk of delay. Though the technical processes could be accelerated, organizational and practical activities could be affected by the complex inclusion of an additional figure in the decision making process.

### “Confusion of the Tongues”

The psychoanalyst Sandor Ferenczi used the expression “confusion of the tongues” to identify the obstacles inherent to communication between adults and children, who are inexorably heterogeneous in their mental representations of relationships and emotional experience. Since then, it has proved an effective expression to refer to interlocutors misinterpreting one another without knowledge.

The expression could be useful when we consider the utilization of AI in medical diagnosis, especially when the latter should be communicated to the patient. The physician is not a simple “translator” of information from the AI to the patient; on the contrary, he or she should play an active role during the process. Let us consider an example: an AI requires that the information on the patient's state is entered according to formats, categories, and languages that it is able to understand and analyze (e.g., data); however, it is possible that not all the relevant information for diagnosis could be transformed as such. How can doctors enter an undefined symptom, a general malaise, or a vague physical discomfort, if the patient himself or herself is hardly able to describe it? Even if trained in the understanding of natural language, the AI will not be able to integrate such information in its original form; this is not related to some sort of malfunction; rather, the AI does not have access to the complex and subtle emotional intelligence abilities that a human doctor can employ when managing a consultation with a patient. Specifically, one risk is that doctors would try to adapt symptoms to AIs' language and capabilities, for example by forcing the information coming from the patient into predefined categories; this could be related to an exaggerated faith in the technology itself, which could lead human users to overestimate its abilities (66).

This could lead patients not being motivated to report doubts, feelings, and personal impressions; indeed, patients can feel when doctors are not really listening to them (67, 68) and could experience a number of negative emotions ranging from anger to demoralization and a sense of abandonment (69–71). Such experiences have a detrimental effect both on the success of shared decision making and on therapy effectiveness because the patient will not adhere to the recommendations (72, 73). In other words, in this way, the new technology would become a source of patient reification, neglecting important elements that only humans' emotional intelligence can grasp.

### Role Ambiguity

When the press started to write about Artificial Intelligence in medicine, a number of authoritative medical sources expressed a firm belief: AI will not replace doctors; it will only help them to do their jobs better, especially by analyzing complex medical data. However, we still have no clear idea about the perception of AIs *on the side of patients*; though it is obvious that AIs will not take over doctors' work, what will the patients think?

According to a recent survey by PwC on 11,000 patients from twelve different countries, 54% of the interviewees were amenable to the idea of being cured by artificial entities, 38% were against it, and rest were uncertain. The highest rates of acceptance can be traced to developing countries, which are open to any innovation in medicine, while countries used to high-level care systems were more critical.

This points to the need for AI innovation to be communicated and explained to patients in the right way, by justifying its added value but also by avoiding the risk that technology takes the place of human doctors *in patients' perception*. For example, as shown by some of the first implementations of the AI system Watson for Oncology by IBM (74, 75), it may happen that the diagnosis provided by AI does not mirror completely the ideas and assessments implemented by the doctor. It is possible that physicians, patients, and AIs will provide different narratives of diagnosis, prognosis, and treatment. This situation of ambiguity and disagreement could lead the patient to experience uncertainty, not knowing what opinion to follow, who really has authority, and who is actually working to help him or her.

When the doctor has to explain the role of AI in the consultation, he or she will have to reassure the patient that the recourse to such a technology is a desirable strategy to employ to provide the best possible consultation and treatment. However, in the perception of the patient, this communication may contain an implicit message, for example, that *someone else* is doing the doctor's work. A recent case was reported in the news worldwide where a patient and his family received a terminal diagnosis from the doctor on a moving robot interface: the family was shocked by the experience and perceived the use of the machine as an insensitive disservice (76).

This is an extreme example where a machine has been introduced in a delicate phase of the healthcare process: even if the machine was not acting autonomously, the effect was disastrous. Obviously, it is fundamental to build an empathic relationship with patients, especially with those dealing with the reality of death and grief (77, 78); while speaking through a machine is “technically the same” as in person, a grieving patient or his caregiver could reasonably feel talking to a robot to be a tragically absurd situation. This example shows how the implementation of technology should be analyzed not only in terms of functionality and technical effectiveness but also from the point of view of patients (79), taking into account their reaction and its consequences for patient health engagement as well as their commitment to shared decision making.

But if the AI is good as my doctor, or maybe even better, whom should I trust? A similar issue exists in medicine already: when multidisciplinary care is offered, patients may experience anxiety and confusion because they have to schedule appointments with several doctors and are not sure who to refer to with specific questions or who to listen to when recommendations are (or appear to them) contradictory (80); patients may find it difficult to trust health providers when the recommendation received is unexpected or counterintuitive (81) and they sometimes consult multiple health professionals, searching for infinite alternative options, as if the cure were some goods to buy, a maladaptive conduct known as “doctor shopping” (82). In other words, when there is disagreement, doubt, or ambiguity in diagnosis and treatment, its effect on the patient's perception and behavior should be taken into consideration and adequately managed within the consultation.

On the side of the doctor, the description of symptoms, diagnosis, and prognosis given by the AI could be more clear and understandable than the patient's; indeed, AI uses medical language and adopts the perspective of a medical professional, relying on objective data and scientific literature. While a patient could often experience difficulties when trying to explain his or her experience, AI could provide a different “narrative” of the diagnosis that the doctor would perceive as more comprehensible and reassuring. In this case, it is possible that the patient's testimony would be undermined or partially ignored, this way losing trace of the nuances and peculiarity of the actual patient's situation, which only a fine-grained analysis of the subjective testimony could detect.

To sum up, AI could potentially take the role of doctors in patients' perception or the opposite.

## Discussion

In this contribution, we tried to identify possible dysfunctional effects of AI's inclusion in medical practice and consultation, conceptualizing them as multiple forms of a “third wheel” effect; besides prefiguring them, it is possible to sketch solutions to the issues to be explored by means of future research.

**Figure 1:**
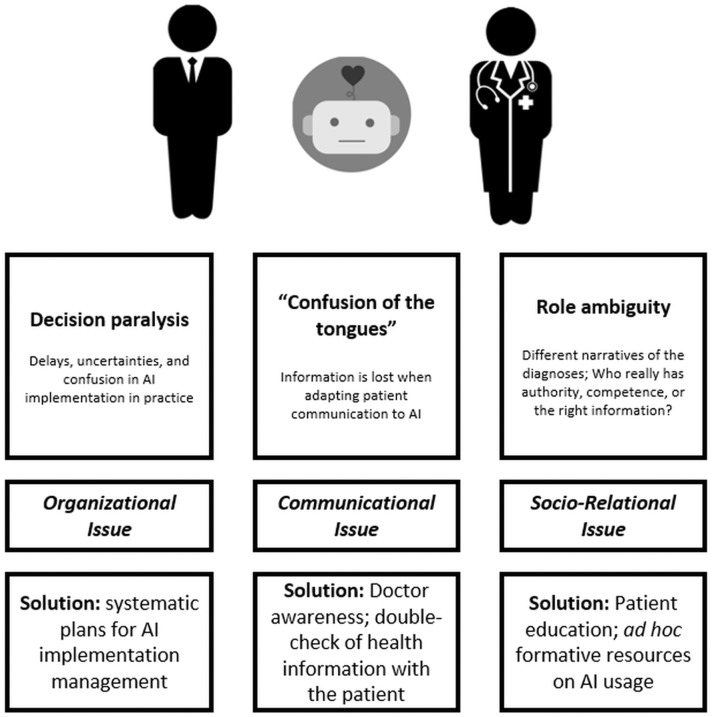
A summary of the “third wheel” effect with possible related solutions.

The three forms of the “third wheel” effect may affect three important areas of the medical consultation: *organizational, communicational*, and *socio-relational* aspects, respectively (see Figure 1 for a summary of the concept).

First, doctors and patients could experience a *decision paralysis*: decisions could be delayed when AIs' recommendations are difficult to understand or to explain to patients. *Decision paralysis* may affect the o*rganizational* aspects of healthcare contexts. It refers to how AI technology will be implemented in healthcare systems that may struggle to adapt their timings, procedures, and organizational boundaries to innovation. Tackling these issues entails making plans for the management of AI implementation that take into consideration not only the benefits of AI for the “technical” aspects of medical activities but also the behavior of organizational units toward AI outcomes and how these outcomes fit among any care practice processes.

Second, the presence of AIs could lead to a “*confusion of the tongues*” between doctors and patients, because patients' health information could be lost or transformed when adapted to AI's classifications. “*Confusion of the tongues”* affects *communication between doctors and patients*. It refers to the actual possibility for patients and doctors to understand each other and enact a desirable process of shared decision making. The solution to these possible issues involves the design of training resources for doctors that make them aware of how AI implementation could be perceived by patients; desirable practices within patient-doctor communication would include double-checking health-related information to address possible confusion arising from the delivery of relevant information mediated by AI.

Lastly, the involvement of AIs could cause confusion regarding roles in patient-doctor relationships when ambiguity or disagreement arises about treatment recommendations. *Role ambiguity* acts on *socio-relational* aspects in healthcare contexts. It refers to the unwanted effects on trust and quality of relationship related to the addition of an artificial interlocutor within the context. These relational aspects are important prerequisites for achieving a desirable healthcare collaboration. Therefore, solutions to role ambiguity issues would entail proper patient education on the usage of AI in their own healthcare journey, especially when intelligent technology resources interact with them directly, mediating treatment (e.g., eHealth). Future studies on the ethical implementation of AI in medical treatment should consider patients' *perception* of these tools and forecast under which conditions patients may feel “put aside” by their doctor because health advice and treatment are delivered by autonomous technologies.

The identification of the psychosocial effects of AI on medical practice is speculative in nature: we should wait until these technologies become actual protagonists in a renovated approach to clinical practice in order to collect data about their effects on the scenario. As a limitation of the present study, we did not report research data; rather, we tried to sketch possible correlates of AI implementation in healthcare based on the literature in health psychology and the social science of technology implementation issues. We believe that consideration of such established phenomena may help pioneers of AI in healthcare to forecast (and possibly manage in advance) issues that will characterize AI implementation as well. Future studies may employ technology acceptance measures to explore health professionals' and patients' attitudes toward artificial intelligence. Moreover, qualitative research methods (e.g., ethnographic observation) could be employed within the pioneer contexts where AIs start to be used in medical consultation, in order to capture the possible obstacles to practice consistent with the third wheel effect prefigured here.

## Author Contributions

ST conceptualized the ideas presented in the article and wrote a first draft. ID helped to refine the theoretical framework and edited the manuscript. GP contributed with important intellectual content and supervised the whole process. All authors contributed to revision, read and approved the submitted version.

## Conflict of Interest

The authors declare that the research was conducted in the absence of any commercial or financial relationships that could be construed as a potential conflict of interest.
